# Supplementary material for: Long-term sea level rise modeling of a basin-tidal inlet system reveals sediment sinks
Source: Nat Commun. 2023 Nov 6;14:7117. doi: 10.1038/s41467-023-42895-y (PMC10628228; doi:10.1038/s41467-023-42895-y)
Supplement: Supplementary file 1 — Supplementary Information [file 41467_2023_42895_MOESM1_ESM.pdf]

nature COMMUNICATIONS

Supplementary Material to:

Long-term sea level rise modeling of a basin and tidal inlet system reveals sediment sinks

Kevin Hanegan[1], Duncan M. FitzGerald[2], Ioannis Y. Georgiou[3,*], Zoe J. Hughes[2]

[1] Moffatt and Nichol, 601 Poydras St, New Orleans LA 70148, USA

[2] Boston University, Department of Earth and Environment, 685 Commonwealth Avenue, Boston MA 02215, USA

[3] The Water Institute of the Gulf, 2021 Lakeshore Dr., Suite 310, New Orleans LA 70122, USA, igeorgiou@thewaterinstitute.org

* Corresponding author

Marsh Evolution

The submergence and loss of backbarrier marsh due to sea-level rise (SRL) is influenced by physical, ecological, and anthropogenic factors. Vertical accretion of a marsh platform is controlled by mineral deposition on the marsh surface, organic below-ground biomass production, and below-ground decomposition [1–3]. SLR in backbarrier marsh environments increases accommodation depth [4] and duration of flooding [5], depending on marsh platform elevation relative to the tidal frame [6,7]. The ability of the marsh to keep pace with this increase in SRL depends on the rate of mineral sedimentation and peat production, which is a function of plant species and platform position within the tidal frame [2,6,8]. Marshes are more resilient in regions of high suspended sediment content (e.g., Winyah Bay, SC [9]), larger tidal ranges [10], and where plants are maintaining their optimum elevation with respect to inundation [2,11,12]. Microtidal settings or areas with low suspended sediment supply experience submergence under even modest rates SLR [10]. The landward extent of many tidal basin saltmarshes are anthropogenically or geologically constrained such that migration inland is not possible [4,13]. In such cases, processes that reduce the lateral extent of marsh platforms, such as edge erosion, lead to irreversible loss of backbarrier marsh area [14–16].

Tidal creeks dissect backbarrier marshes, acting as important conduits for water, nutrients and sediment [17]. The drainage density of creek networks and the cross-sectional area of individual channels is positively correlated to the tidal prism of the basin as a whole and the local prism of individual creeks [18,19]. SLR increases tidal prism directly by adding to the cross-sectional area of the flow, as well as flooding backbarrier marsh platforms more frequently and to deeper depths [20]. Higher discharge through the drainage network increases currents and shear stress which leads to incision of the tidal creeks and bank erosion, thus maintaining the local equilibrium relationship between tidal prism and creek cross-sectional area. Moreover, increased shear stresses along channel sides may promote new tributary initiation and headward erosion that jointly further expand the creek network [9]. Additionally, rapid headward erosion of tidal channels may be aided by vegetation collapse and bioturbation [21]. Channel network incision and expansion liberates sediment that becomes available for deposition on adjacent marsh surfaces [22,23].

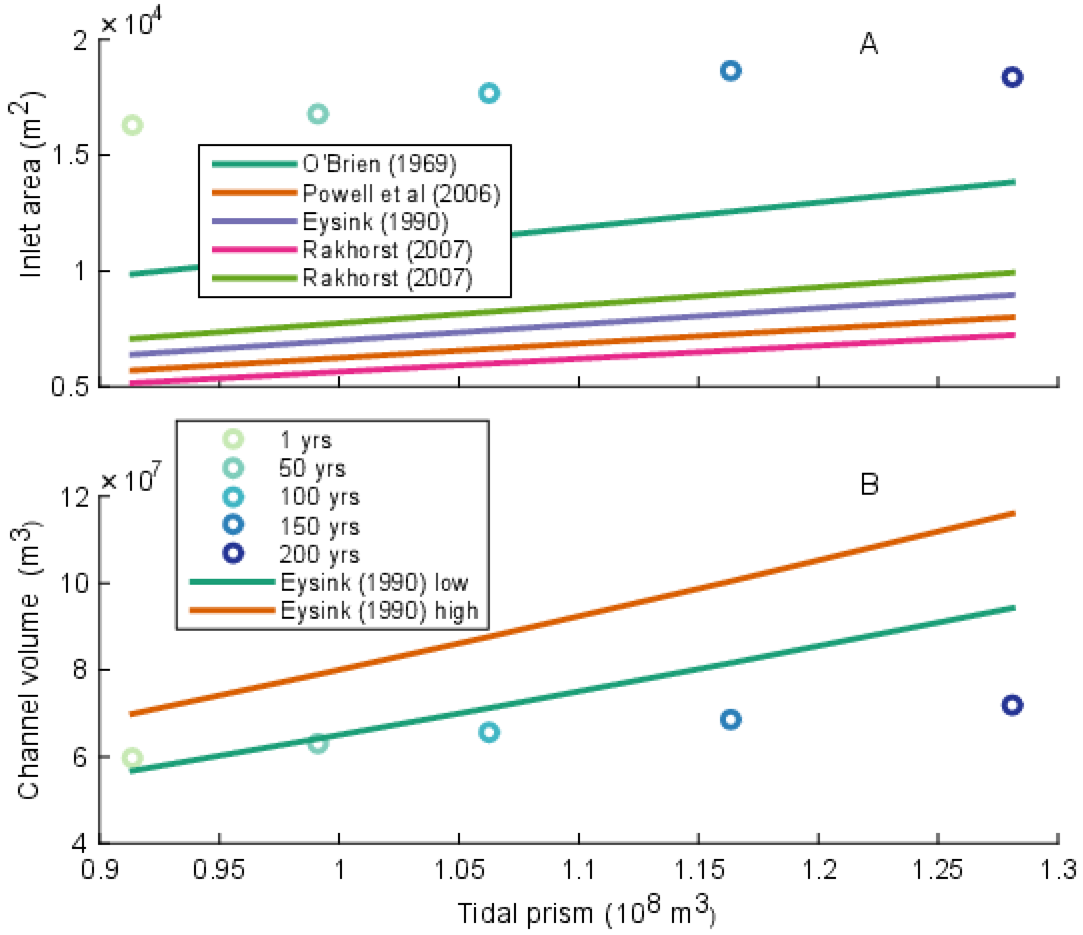

Supplementary Fig. 1 | Evaluation of inlet and channel geometries through time. (A) Tidal prism versus inlet cross-sectional area using Stive & Rakhorst (2008) empirical coefficients and (B) Tidal prism versus total channel volume below the mean lower water using the high and low coefficients for Dutch tidal basins reported in Eysink (1990).

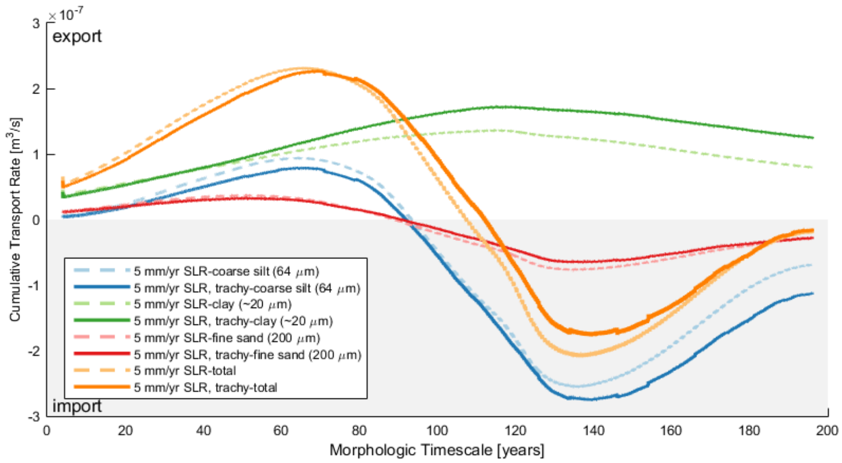

Supplementary Fig. 2 | Cumulative sediment transport (total and for individual sediment fractions) rates through the inlet for the 5 mm/yr SLR case, with and without trachytope vegetation-enhanced bottom roughness[24]. Positive values indicate sediment export while negative values indicate sediment import. The case with vegetation-enhanced bottom roughness is represented by solid lines while the simulation without trachytope roughness uses lighter, dashed lines of the same color. The cumulative transport time series has been filtered to remove tidal fluctuations so that the plotted rates reflect residual transport. Impacts of vegetation-enhanced bottom roughness on cumulative sediment transport are minimal for this case.

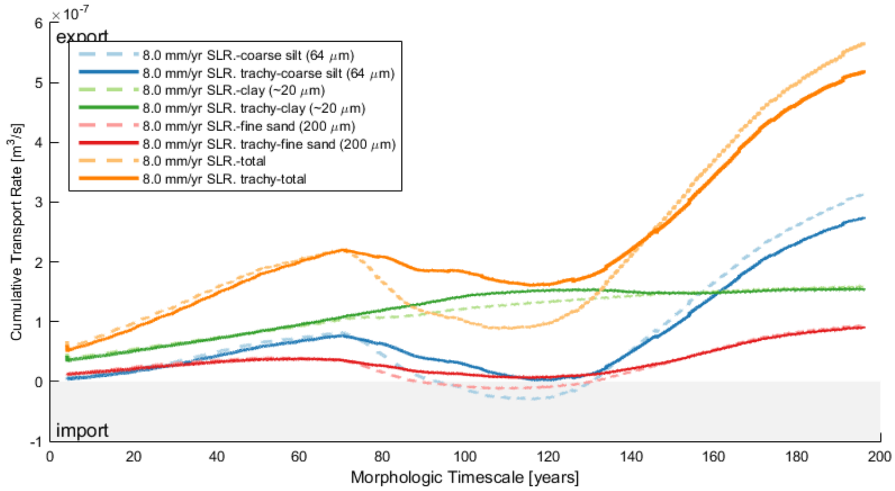

Supplementary Fig. 3 | Cumulative sediment transport (total and for individual sediment fractions) rates through the inlet for the 8 mm/yr SLR, 3 mm/yr marsh accretion case, with and without trachytope vegetation-enhanced bottom roughness[24]. Positive values indicate sediment export while negative values indicate sediment import. The case with vegetation-enhanced bottom roughness is represented by solid lines while the simulation without trachytope roughness uses lighter, dashed lines of the same color. The cumulative transport time series has been filtered to remove tidal fluctuations so that the plotted rates reflect residual transport. Enhanced bottom roughness slightly increases ebb currents during the middle portion of the simulation (approximately years 80 through 140) which increases export of coarse sediments relative to the simulation with no trachytope roughness, though flux trends outside of this period are minimally impacted.

Conceptualize Basin Comparison

We envision the conceptual basin being like those along many mixed-energy barrier island coasts (see Table 1) such as Plum Island Sound Inlet in northern Massachusetts. As viewed in the vertical aerial photograph in Fig. S4a, this system is fronted by a well-formed ebb-tidal delta and contains an elongate backbarrier with numerous shoals and surrounding marsh. Time series at hydrographic stations #1-3 (Fig. S4b) illustrate that tidal elevations along the length of Plum Island Sound exhibit a standing wave condition with little tidal lag or dampening. A close-up view of Middle Ground (see Fig. S4a) indicates extensive long-term influx of sand and shoal formation (Fig. S5).

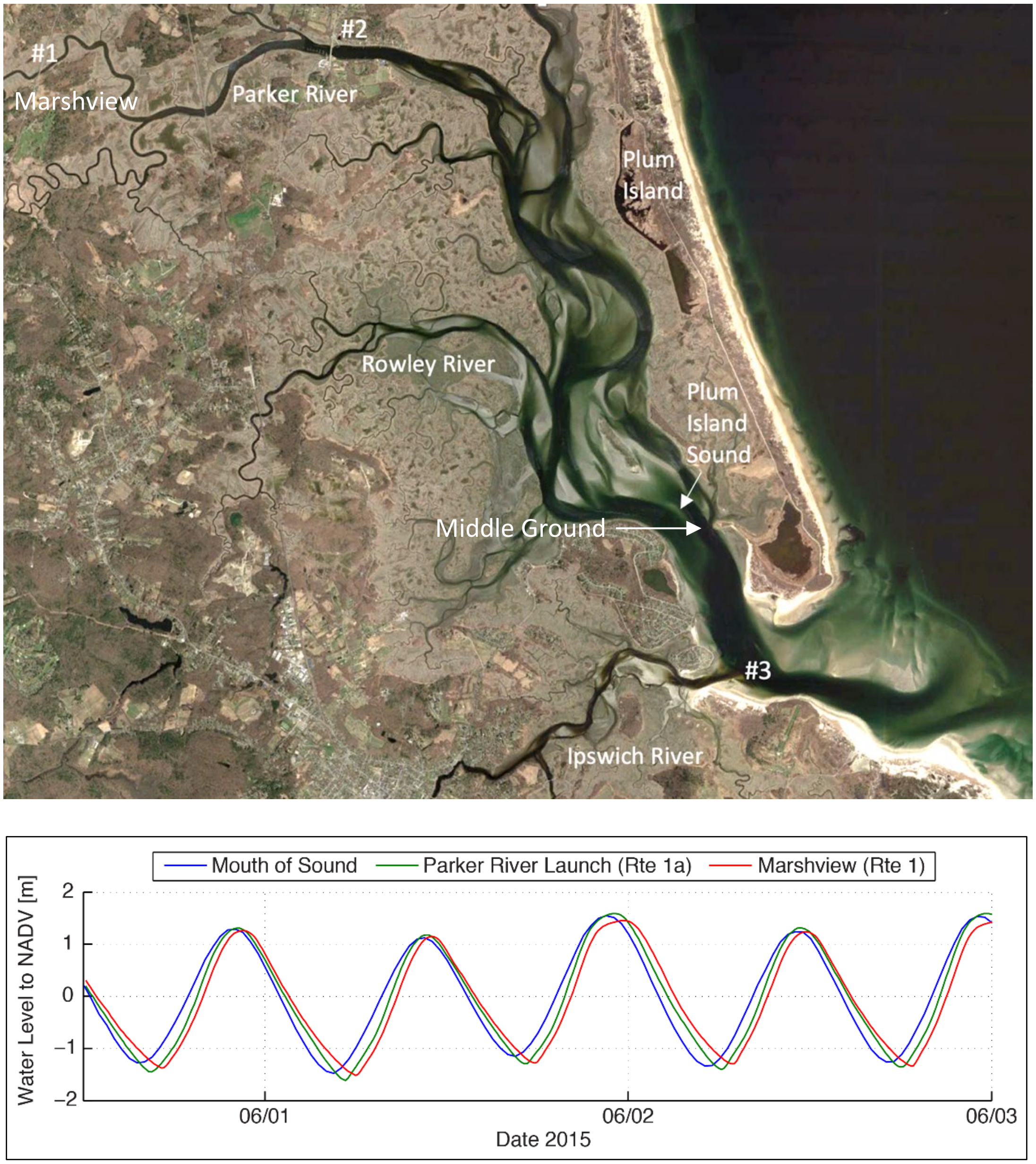

B

Supplementary Fig. 4 | Plum Island Sound, Massachusetts. A. Low-tide aerial photo showing Sound is floored by numerous sand shoals. Numbers refer to hydrographic stations. B. Note that time series reveal little dampening of the tidal wave or lag of high and low water.

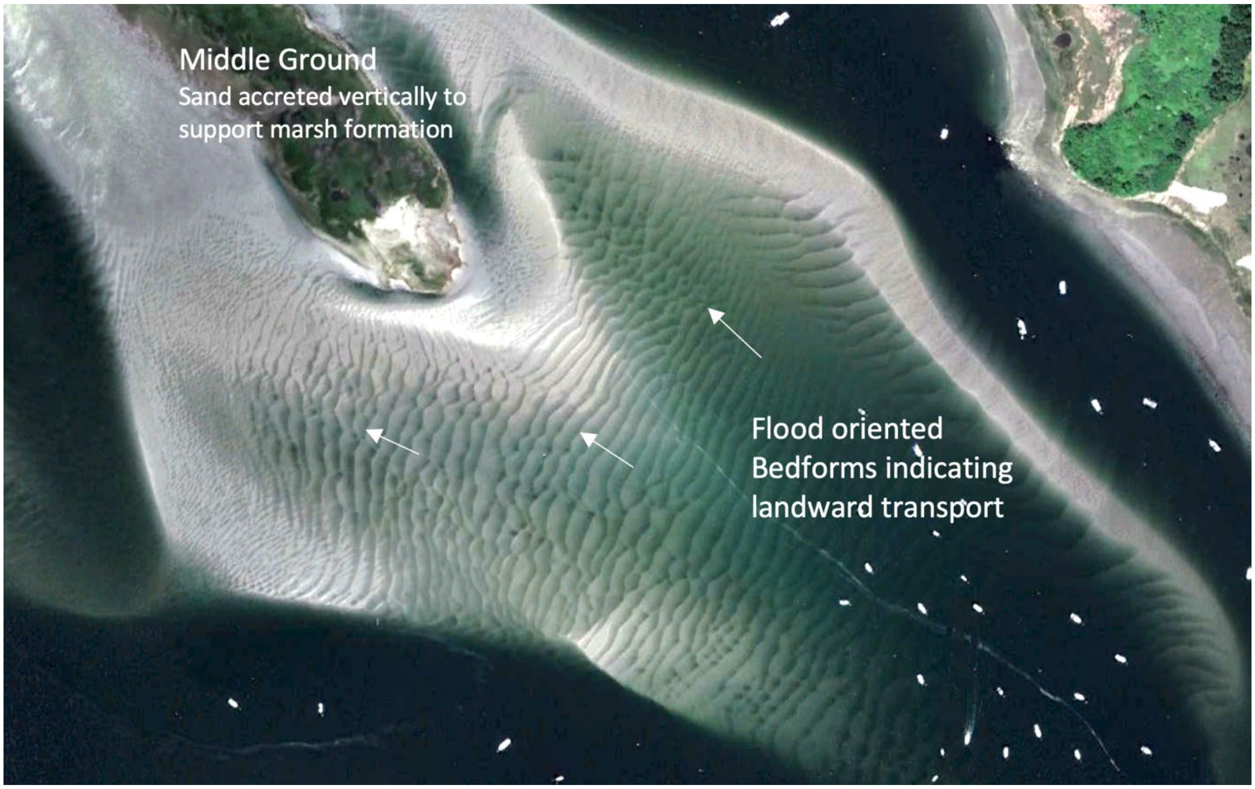

Supplementary Fig. 5 | Sand movement into the Sound and accumulating on the flood-tidal delta indicated by flood-oriented bedforms and construction of saltmarsh on vertically accreting shoal.

Inclusion of Waves in Model

Our modeled basin contains a relatively narrow open-water backbarrier with numerous shoals and thus, the impact of waves on sediment transport is limited and localized. The ebb-tidal delta and adjacent shoreline are areas where waves augment sediment transport processes. Our modeling results of variable conditions show that waves produce limited net sedimentological change in the basin. Using a scenario that simulates 100 years and imposing a 5 mm/yr SLR, identical to a previous scenario, and using a schematized wave with an oblique wave approach (75-degree), a 6-second period, and 0.25-m wave height, we illustrate typical model results in Figure S6c. As seen, the seaward portion of the ebb-tidal delta undergoes uniform erosion while the region inside the inlet experiences areas of slight erosion and deposition. Widening of the inlet is offset by slight deposition at the inlet throat. While waves cause re-organization of the backbarrier basin, when compared to the scenarios with no waves (Fig. 6a, b), it is evident that the order of change in morphology is similar. For example, the channel incision remains in the same pathway with small changes in patterns of erosion and deposition attributed to differences in lateral migration. Furthermore, waves have a small influence on the tidal flats (Fig S6c), but when compared to the scenarios without waves (Fig. S6a, b) the patterns and magnitude of change lie within the two scenarios without significant changes in the basin hypsometry. Thus, adding waves does not change the overall conclusions.

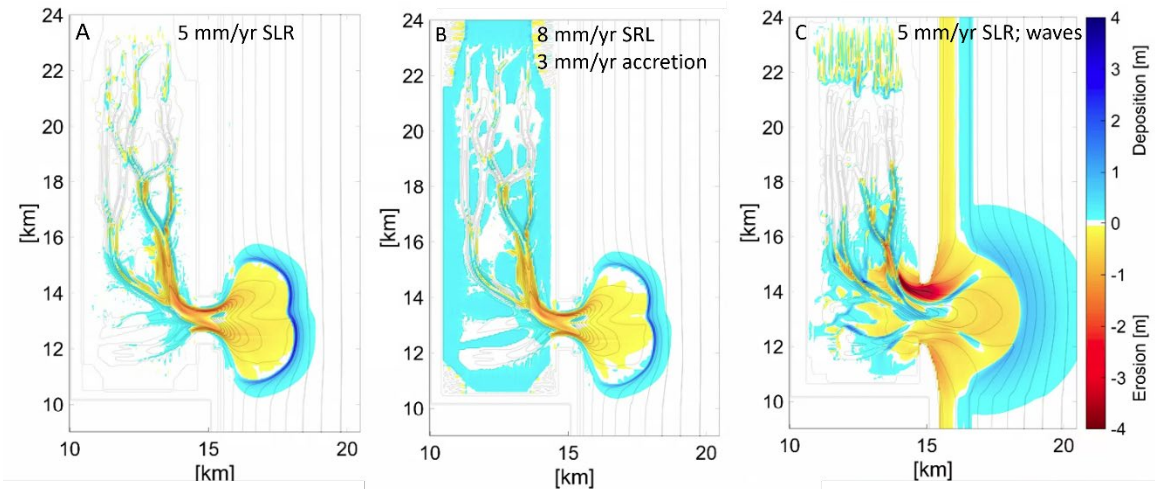

Supplementary Fig. 6 | Model showing results with and without waves A. no waves, SLR = 5mm/yr, B. no waves SLR = 8 mm/yr with marsh accretion of 3 mm/yr, and C. influence of waves with SLR = 5 mm/yr.

Changes in Clay Content

Increasing and decreasing the clay content of the modeled sediment by 10 percent to 24% and 4% (by adjusting the sand content), respectively provides a means of investigating the impact of clay abundance (Fig. S7). The model results suggest that sand and silt exhibit the same long-term trend of export followed by import as the original 14% clay content (Fig. 4), while the magnitude of net erosion/accumulation is slight. In contrast, while clay is continuously exported in all cases, the volume of clay discharged from the basin, as expected, is commensurate with the initial clay content.

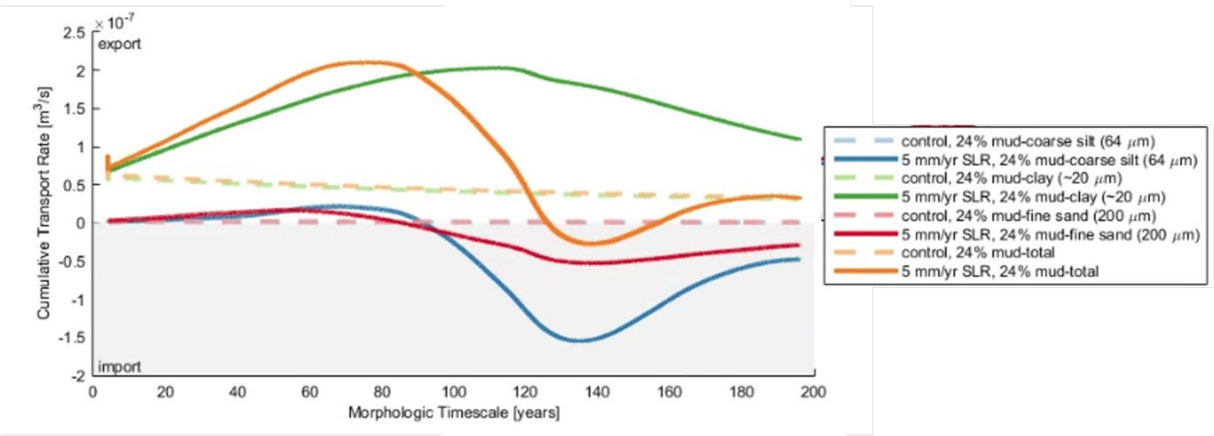

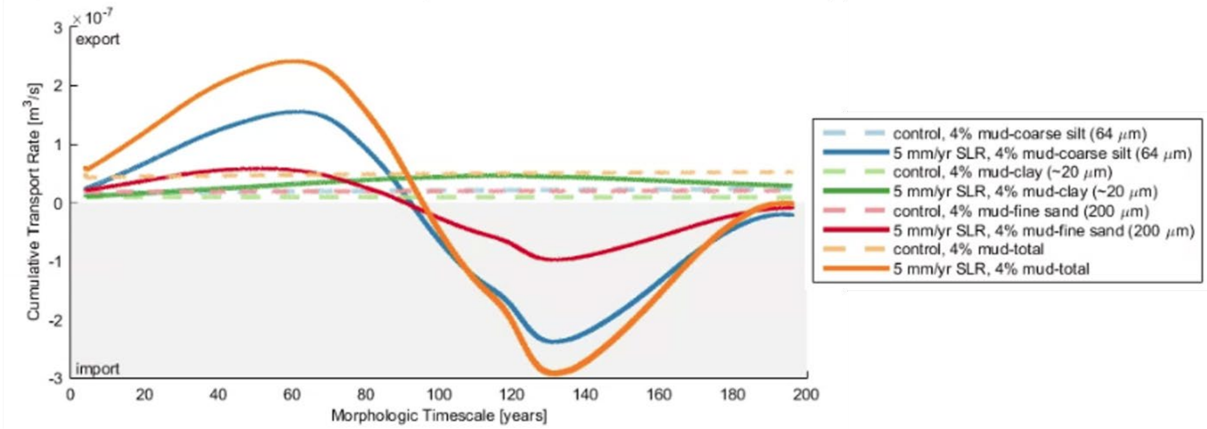

Supplementary Fig. 7 | Model results of clay content of 24% (top) and 4% (bottom). Lines show trends for silt (blue), clay (green), sand (red), and total (orange).

The Vegetation parameters used in the vegetation sensitivity testing which are input to the Baptist formulation are shown in Table SI Tables S1.

Supplementary Table 1 | Vegetation Information for Baptist Equation

Parameter	Value
Stem Height [m]	0.5
Vegetation Density [stems/m]	0.05
Stem Density [stems/m2]	185
Drag Coefficient	1
Alluvial Bed Roughness [m^(1/2)/s]	60

Sediment Parameters

The sediment parameters, including critical shear stress for erosion and erosion rate, we used in the Parteniades-Krone equation for fine sediment transport are provided in SI Table 2.

Supplementary Table 2 | Sediment characteristics and other sediment and morphology relevant parameters

Category	Parameter	Values
**Sediment characteristics**	Sand median grain size (D50)	200 µm
	Coarse silt median grain size (D50)	64 µm
	Settling velocity (clay fraction)	0.25 mm/s (velocities correspond to D50s of 20 µm, respectively)
	Critical shear stress for erosion of consolidated mud (i.e., clay)	0.5 Pa
	Dry bed (or bulk) density of consolidated mud (i.e., silt and clay)	500 kg/m^3
	Dry bed (or bulk) density of sand	1600 kg/m^3
**Sediment transport**	Erosion parameter	0.0001 Kg/m^2/s
	Current related reference concentration factor/transport magnitude factor	1 / 1
**Morphology**	Mobile bed composition	43% sand / 43% silt / 14% clay<br>Sensitivity with<br>53% sand / 43% silt / 4% clay<br>33% sand / 43% silt / 24% clay
	Morphological scale factor (MorFac) for cold-front simulations	100
	Dry cell erosion factor	1

Supplementary Material References

1. Benner, R., Fogel, M. L. & Sprague, E. K. Diagenesis of belowground biomass of Spartina alterniflora in salt-marsh sediments. *Limnol Oceanogr* **36**, 1358–1374 (1991).
2. Morris, J. T., Sundareshwar, P. V., Nietch, C. T., Kjerfve, B. & Cahoon, D. R. Responses of Coastal Wetlands to Rising Sea Level. *Ecology* **83**, 2869–2877 (2002).
3. Valiela, I. *et al.* Decomposition in salt marsh ecosystems: The phases and major factors affecting disappearance of above-ground organic matter. *J Exp Mar Biol Ecol* **89**, 29–54 (1985).
4. Törnqvist, T. E., Cahoon, D. R., Morris, J. T. & Day, J. W. Coastal Wetland Resilience, Accelerated Sea-Level Rise, and the Importance of Timescale. *AGU Advances* **2**, e2020AV000334 (2021).
5. Snedden, G. A., Cretini, K. & Patton, B. Inundation and salinity impacts to above-and belowground productivity in Spartina patens and Spartina alterniflora in the Mississippi River deltaic plain: Implications for using river diversions as restoration tools. *Ecol Eng* **81**, 133–139 (2015).
6. Reed, D. J. The response of coastal marshes to sea-level rise: Survival or submergence? *Earth Surf Process Landf* **20**, 39–48 (1995).
7. FitzGerald, D. M. *et al.* Largest marsh in New England near a precipice. *Geomorphology* **379**, 107625 (2021).
8. Mudd, S. M., D'Alpaos, A. & Morris, J. T. How does vegetation affect sedimentation on tidal marshes? Investigating particle capture and hydrodynamic controls on biologically mediated sedimentation. *J Geophys Res* **115**, F03029 (2010).
9. D'Alpaos, A. Tidal network ontogeny: Channel initiation and early development. *J Geophys Res* **110**, F02001 (2005).
10. Kirwan, M. L. *et al.* Limits on the adaptability of coastal marshes to rising sea level. *Geophys Res Lett* **37**, (2010).
11. Mudd, S. M. The life and death of salt marshes in response to anthropogenic disturbance of sediment supply. *Geology* (2011).
12. Kirwan, M. L. & Temmerman, S. Coastal marsh response to historical and future sea-level acceleration. *Quat Sci Rev* **28**, 1801–1808 (2009).
13. Schile, L. M. *et al.* Modeling tidal marsh distribution with sea-level rise: evaluating the role of vegetation, sediment, and upland habitat in marsh resiliency. *PLoS One* **9**, e88760 (2014).
14. Mariotti, G. & Fagherazzi, S. Critical width of tidal flats triggers marsh collapse in the absence of sea-level rise. *Proc Natl Acad Sci U S A* **110**, 5353–6 (2013).
15. Leonardi, N., Ganju, N. K. & Fagherazzi, S. A linear relationship between wave power and erosion determines salt-marsh resilience to violent storms and hurricanes. *Proc Natl Acad Sci U S A* **113**, 64–8 (2016).
16. Li, X., Leonardi, N. & Plater, A. J. Wave-driven sediment resuspension and salt marsh frontal erosion alter the export of sediments from macro-tidal estuaries. *Geomorphology* (2019) doi:10.1016/j.geomorph.2018.10.004.

17. Hughes, Z. J. Tidal Channels on Tidal Flats and Marshes. in *Principles of Tidal Sedimentology* (eds. Davis Jr., R. A. & Dalrymple, R. W.) 269–300 (Springer Netherlands, 2012). doi:10.1007/978-94-007-0123-6_11.
18. D'Alpaos, A., Lanzoni, S., Marani, M. & Rinaldo, A. On the tidal prism–channel area relations. *J Geophys Res* **115**, F01003 (2010).
19. Eysink, W. D. Morphologic response of tidal basins to changes. *Coastal Engineering Proceedings* **1**, (1990).
20. FitzGerald, D. M., Fenster, M. S., Argow, B. A. & Buynevich, I. V. Coastal Impacts Due to Sea-Level Rise. *Annu Rev Earth Planet Sci* **36**, 601–647 (2008).
21. Hughes, Z. J. *et al.* Rapid headward erosion of marsh creeks in response to relative sea level rise. *Geophys Res Lett* **36**, L03602 (2009).
22. Hopkinson, C. S., Morris, J. T., Fagherazzi, S., Wollheim, W. M. & Raymond, P. A. Lateral Marsh Edge Erosion as a Source of Sediments for Vertical Marsh Accretion. *J Geophys Res Biogeosci* **123**, 2444–2465 (2018).
23. Houttuijn Bloemendaal, L. J. H., FitzGerald, D. M., Hughes, Z. J., Novak, A. B. & Phippen, P. What controls marsh edge erosion? *Geomorphology* **386**, 107745 (2021).
24. Baptist, M. J. Modelling floodplain biogeomorphology. *Delft University of Technology, Delft, Netherlands* (2005).
